# Indoxyl Sulfate-Mediated Blood–Brain Barrier Damage in Chronic Kidney Disease

**DOI:** 10.3390/toxins18080334

**Published:** 2026-08-01

**Authors:** Leah Hernandez, Camillo Tancredi Strizzi, Miriam Rosina, Angelina Schwarz, Nina Kronqvist, Samsul Arefin, Peter Stenvinkel, Karolina Kublickiene

**Affiliations:** 1 Division of Renal Medicine, Clinical Science, Intervention and Technology (CLINTEC), Karolinska Institutet, 141 52 Huddinge, Sweden; camillo.tancredi.strizzi@ki.se (C.T.S.); angelina.schwarz@ki.se (A.S.); samsul.arefin@ki.se (S.A.); peter.stenvinkel@ki.se (P.S.); 2 Department of Translational Medicine and Surgery, Università Cattolica del Sacro Cuore, 00168 Rome, Italy; 3 Nephrology, Dialysis and Transplantation Unit, Fondazione Policlinico Universitario A. Gemelli IRCCS, 00168 Rome, Italy; 4 Department of of Translational Medicine (DIMET), University of Piemonte Orientale (UPO), 28100 Novara, Italy; miriam.rosina@uniupo.it; 5 Department of Medicine, Karolinska Institutet, 141 83 Huddinge, Sweden; nina.kronqvist@ki.se

**Keywords:** indoxyl sulfate, chronic kidney disease, blood-brain barrier, uremic toxin

## Abstract

Chronic kidney disease is associated with neurovascular complications including cognitive impairment, potentially involving blood–brain barrier (BBB) impairment. The protein-bound uremic toxin indoxyl sulfate (IS) promotes endothelial injury, but its effects on human brain microvascular endothelial cells remain incompletely understood. Human cerebral microvascular endothelial cells (hCMEC/D3) were exposed to IS 200 and 900 μM. BBB integrity was assessed by the FITC-dextran (4 kDa) transwell permeability assay and claudin-5 immunofluorescence. Transcriptional responses were quantified by qPCR for aryl hydrocarbon (AhR) target genes, oxidative stress-associated and inflammatory markers, senescence, and junction-associated genes. Senescence-associated phenotypic changes were evaluated by SA-β-galactosidase staining and cytokine array profiling. IS increased endothelial permeability at 24 and 48 h (~1.5-fold relative to control) without evidence of cytotoxicity and reduced claudin-5 staining intensity. IS strongly upregulated AhR target genes, including CYP1A1, CYP1B1, and CYP1A2. NFE2L2 and IDO1 increased, while NFKB1 remained unchanged. SA-β-gal positivity increased, accompanied by elevated GM-CSF and G-CSF secretion, while CDKN1A decreased at IS 900 µM and CDKN2A remained unchanged. CDH5 was downregulated, TJP1 increased at 900 µM, and CLDN5 remained unchanged. These findings indicate that IS exposure is associated with impaired BBB integrity, AhR-related transcriptional responses, oxidative stress-associated transcriptional changes, junctional remodeling, and senescence-like endothelial features. However, causal attribution to individual pathways requires inhibition or knockdown studies.

## 1. Introduction

Chronic kidney disease (CKD) affects approximately 850 million people worldwide and is associated with significant morbidity and mortality [1]. Progressive loss of kidney function leads to retention of protein-bound uremic toxins, among which indoxyl sulfate (IS) has been implicated in microvascular endothelial dysfunction and cardiovascular disease [2,3]. IS is poorly cleared by conventional dialysis and promotes endothelial injury through pro-oxidant, pro-inflammatory, and pro-senescent mechanisms [4,5].

CKD is increasingly recognized as a model of early vascular aging (EVA), characterized by premature arterial stiffening, endothelial dysfunction, and a biological age exceeding chronological age [5,6,7,8]. At the cellular level, EVA in CKD is driven by the accumulation of senescent cells expressing a senescence-associated secretory phenotype (SASP), which sustains a pro-inflammatory milieu and promotes further vascular injury [9]. Uremic toxins, including IS, are thought to accelerate this process by activating the aryl hydrocarbon receptor (AhR) and its downstream cytochrome P450 targets, leading to generation of reactive metabolites and oxidative stress in the endothelium [10,11,12].

Beyond cardiovascular complications, CKD is increasingly recognized as a risk factor for cognitive impairment and stroke [13]. These neurological manifestations have been attributed, at least in part, to compromised blood–brain barrier (BBB) function [14,15] although the underlying mechanisms remain poorly understood. The BBB is a highly selective barrier that maintains central nervous system solute balance [16] by restricting the passage of potentially harmful substances from systemic circulation into the brain parenchyma [16]. This barrier function is primarily mediated by endothelial cells which are interconnected by tight junction proteins such as claudin-5, occludin and JAM-1 [17].

Previous studies have linked IS accumulation and AhR activation to BBB disruption and cognitive impairment in an experimental rodent CKD model [18]. IS has also been shown to alter oxidative stress- and nitric oxide-related responses in murine cerebral endothelial cells [19]. In addition, IS-associated endothelial dysfunction has also been described in peripheral human microvascular endothelial cells and human resistance-vessel models [4,20]. However, the combined assessment of IS-associated permeability changes, junctional gene expression, AhR-related transcriptional changes, senescence-like endothelial features, and secretome alterations has, to our knowledge, not been characterized in a human cerebral microvascular endothelial model. Our study addresses this gap using hCMEC/D3 cells within the EVA framework of CKD.

We hypothesize that IS concentrations selected to reflect reported CKD-associated exposure ranges would be associated with impaired BBB integrity in human cerebral microvascular endothelial cells. Using hCMEC/D3 monolayers, we examined whether IS exposure: (1) increases endothelial permeability; (2) disrupts endothelial junction organization; (3) alters transcriptional responses related to inflammation, oxidative stress, and senescence; and (4) induces cellular senescence.

## 2. Results

### 2.1. Indoxyl Sulfate Does Not Induce Overt Toxicity in hCMEC/D3 Cells

**Figure 1:**
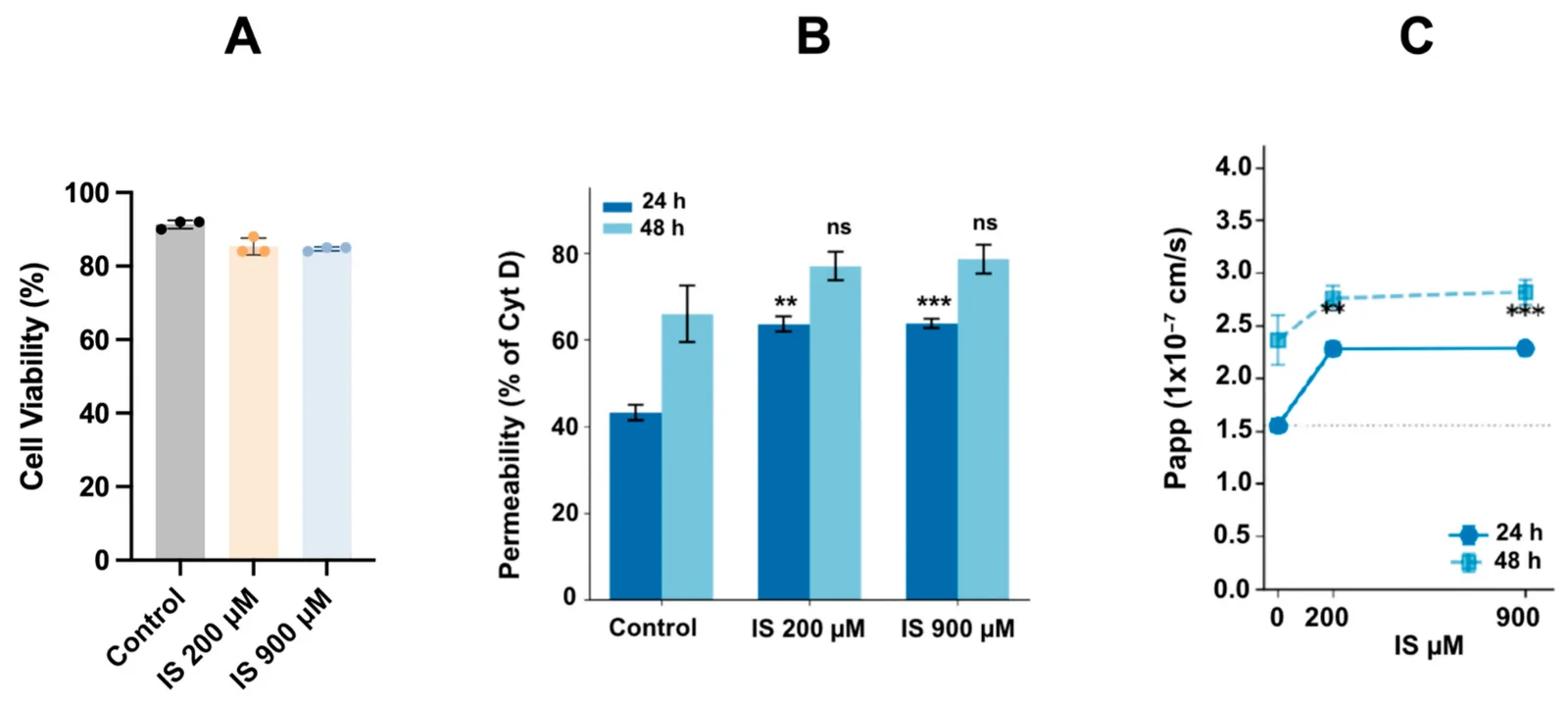
(**A**) Cell viability of hCMEC/D3 after 48 h IS exposure. No statistically significant difference observed between control and IS-treated cells. (**B**) Percent permeability at 24 and 48 h for control, IS (200 and 900 µM), relative to cytochalasin D (positive control). Permeability increased over time across all conditions, indicating a time-dependent increase in barrier permeability. (**C**) Dose–response of apparent permeability (Papp) as a function of IS concentration at 24 h and 48 h. Papp showed a plateau in permeability response. Data are presented as mean ± SD from n = 3 independent biological experiments. Statistical comparisons were performed using a mixed-effect model with Šídák’s multiple-comparison test. Statistical significance is indicated as ** *p* < 0.01, *** *p* < 0.001, ns (non-significant).

To determine if IS alters endothelial cell viability, hCMEC/D3 monolayers were treated with IS (200 or 900 µM) for 48 h. Cell viability remained above 80% in all conditions, with no statistically significant differences between groups (Figure 1A). These findings indicate that subsequent functional and transcriptional changes are not attributable to cell loss.

### 2.2. Indoxyl Sulfate Increases Endothelial Barrier Permeability

To assess the IS-induced functional changes in the endothelial barrier, hCMEC/D3 monolayers were exposed to IS at concentrations spanning the uremic range. Permeability was expressed relative to the Cytochalasin D positive control, representing maximal barrier disruption in this assay.

At 24 h, IS 200 µM increased in endothelial permeability compared with control. No further increase in permeability was observed at 900 µM, indicating a plateau in the permeability response at this timepoint.

At 48 h, IS-treated monolayers continued to show higher permeability compared to controls. Although baseline permeability values increased over time in all conditions, consistent with known time-dependent drift in hCMEC/D3 monolayers, IS-treated cells maintained consistently higher permeability than controls at both timepoints (Figure 1B,C; Papp values across all conditions are shown in Figure S1).

### 2.3. Indoxyl Sulfate Reduces Claudin-5 Staining Intensity

**Figure 2:**
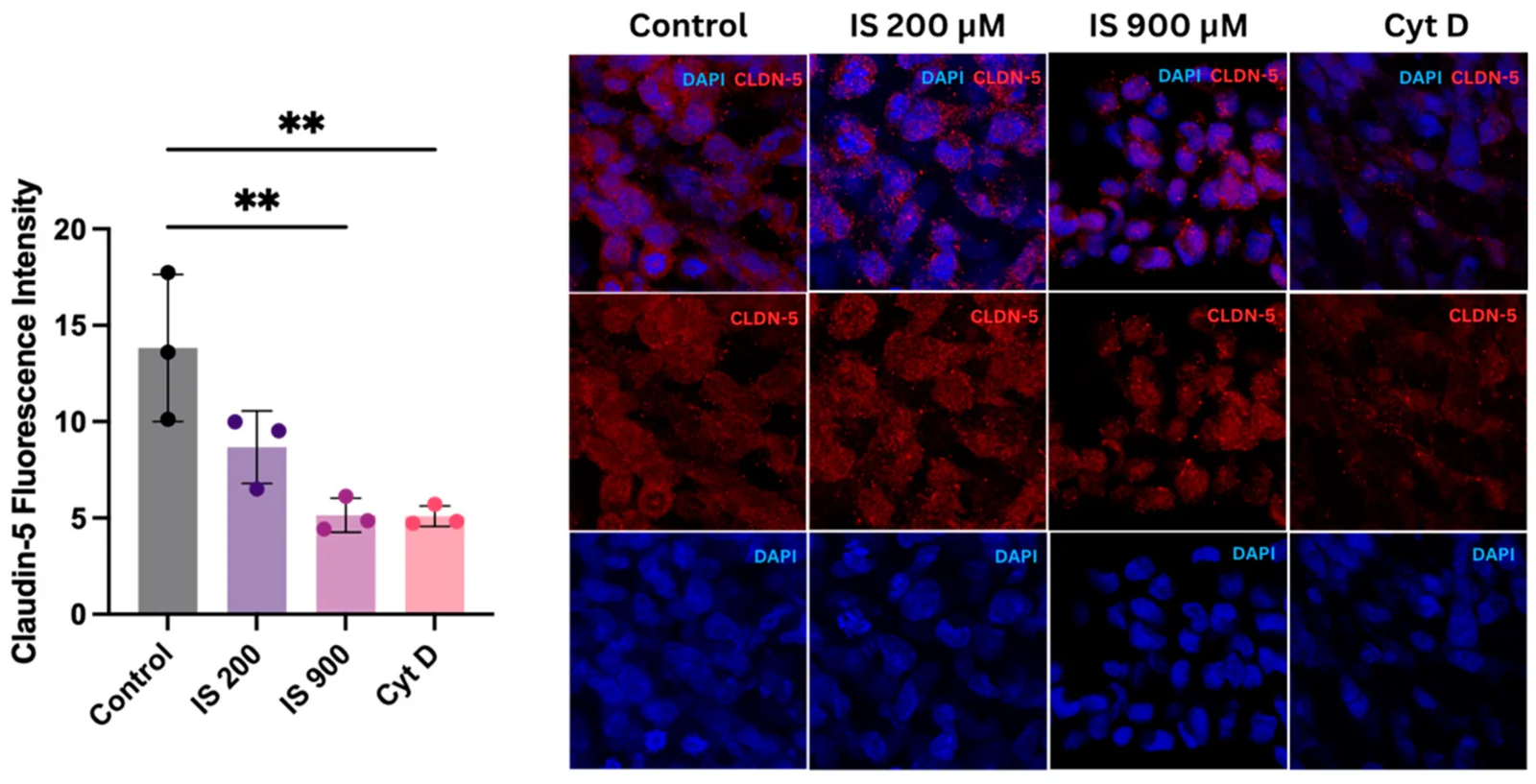
Quantification of fluorescence intensity from four fields per insert and averaged per insert (n = 3 independent experiments; mixed-effect model with Šídák’s multiple-comparison test). Representative images show claudin-5 staining (red) and DAPI nuclear staining (blue) in control, IS 200 µM, IS 900 µM, and Cytochalasin D-treated cells. Separate CLDN-5 and DAPI channels are shown below the merged images. Abbreviation: IS (indoxyl sulfate), Cyt D (Cytochalasin D), CLDN-5 (Claudin-5), AU (arbitrary units), DAPI (4′, 6-diamidino-2-phenylindole). Data presented as mean ± SD. Statistical significance is indicated as ** *p* < 0.01.

Immunofluorescence quantification of claudin-5 showed a reduction in signal intensity in IS-treated cells compared to control, particularly at 900 µM concentration. Cytochalasin D positive control showed the expected reduction in claudin-5 staining intensity compared with control. These findings suggest a possible disruption in claudin-5 localization or abundance following IS exposure, but require confirmation with a larger number of independent replicates (Figure 2).

### 2.4. Indoxyl Sulfate Activates AhR Signaling

**Figure 3:**
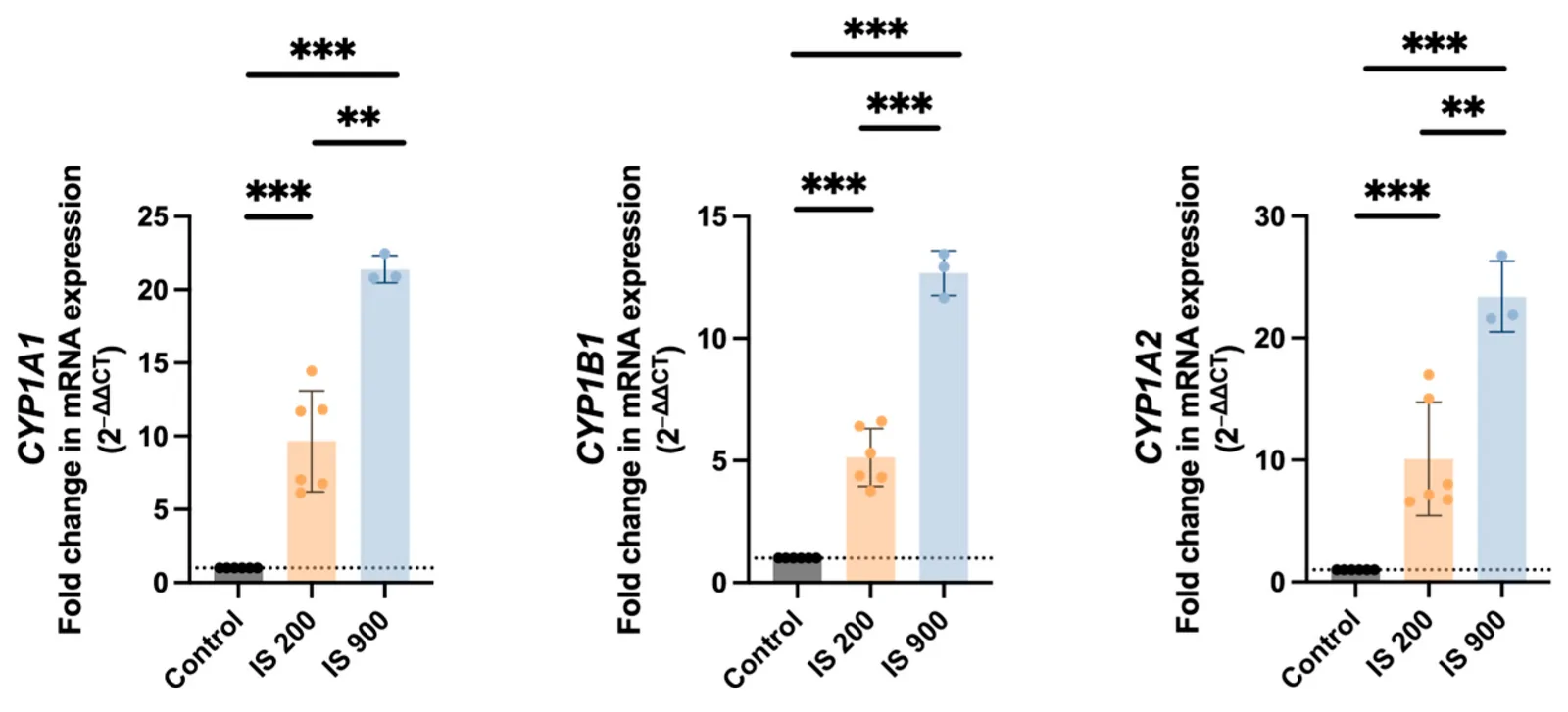
Relative mRNA expression of AhR target genes (*CYP1A1*, *CYP1B1*, *CYP1A2*) in hCMEC/D3 cells exposed to IS (200 and 900 µM) for 48 h, expressed as fold-change relative to control. Data presented as mean ± SD (n = 6 independent experiments for IS 200 µM; n = 3 independent experiments for IS 900 µM). Statistical comparisons performed using a mixed-effect model with Šídák’s multiple-comparison test. The dashed horizontal line indicates the baseline control expression (fold change = 1). Statistical significance is indicated as ** *p* < 0.01, *** *p* < 0.001. Abbreviations: Cyp (Cytochrome), IS (indoxyl sulfate).

Exposure to IS resulted in a dose-dependent upregulation of canonical AhR target genes. At 200 µM, CYP1A1, CYP1B1, and CYP1A2 expression increased approximately 10-fold, 5-fold, and 10-fold, respectively, compared to control. At 900 µM, expression was further elevated to approximately 20–24-fold (CYP1A1, CYP1A2) and 12-fold (CYP1B1) (Figure 3). These data indicate robust, concentration-dependent induction of AhR target genes in brain endothelial cells following IS exposure. Whether the activation is mechanistically required for the observed barrier and junctional changes was not directly tested in this study as no AhR antagonist experiment was performed.

### 2.5. Indoxyl Sulfate Selectively Alters Oxidative Stress-Related, Inflammatory, and Senescence-Associated Transcriptional Responses

To assess whether IS exposure was associated with broader transcriptional changes beyond AhR signaling, the expression of genes associated with oxidative stress, inflammation, and senescence was quantified following treatment with IS (200 and 900 µM).

**Figure 4:**
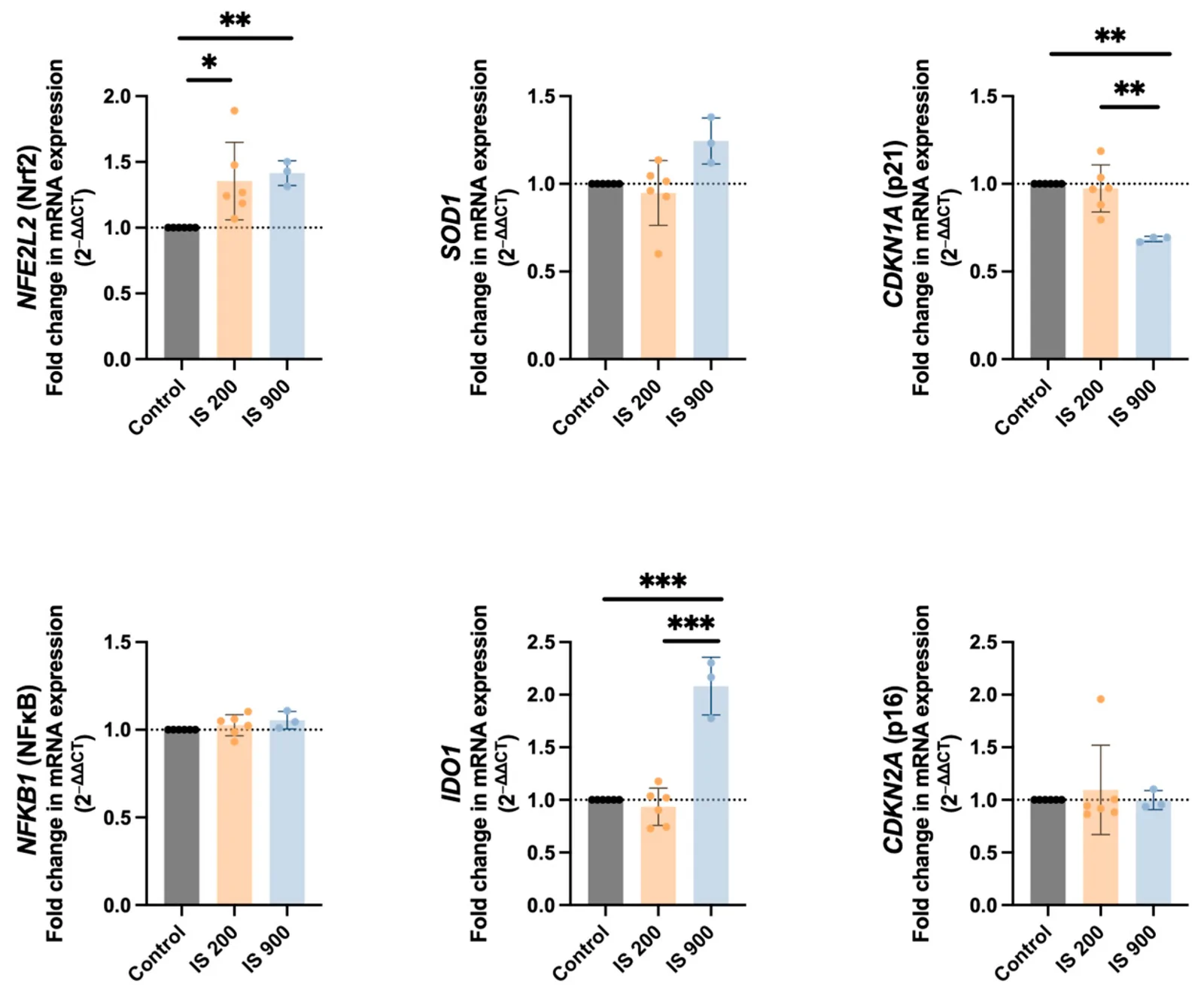
Relative mRNA expression of genes associated with oxidative stress, inflammation and senescence (NFE2L2, SOD1, NFKB1, IDO1, CDKN1A, and CDKN2A). Data presented as mean ± SD (n = 6 independent experiments for IS 200 µM; n = 3 independent experiments for IS 900 µM). Statistical comparisons performed using a mixed-effect model with Šídák’s multiple-comparison test. The dashed horizontal line indicates the baseline control expression (fold change = 1). Statistical significance is indicated as * *p* < 0.05, ** *p* < 0.01, *** *p* < 0.001. Abbreviations: NFE2L2 (nuclear factor erythroid 2-related factor 2), SOD1 (superoxide dismutase 1), NFκB (nuclear factor kappa B1), IDO1 (indoleamine 2,3-dioxygenase 1), CDKN1A (cyclin-dependent kinase inhibitor 1A), CDKN2A (cyclin-dependent kinase inhibitor 2A), IS (indoxyl sulfate).

NFE2L2, which encodes the antioxidant stress-response transcription factor Nrf2, was significantly upregulated at both IS 200 µM and IS 900 µM concentrations compared with control. SOD1 expression was not significantly altered following IS exposure. NFKB1, which encodes NFκB, remained unchanged, whereas IDO1 was significantly upregulated at IS 900 µM compared with both control and IS 200 µM (Figure 4).

For senescence-associated genes, CDKN1A expression was significantly reduced at IS 900 µM compared with control and IS 200 µM, while CDKN2A remained near control levels and was not significantly altered (Figure 4).

### 2.6. Indoxyl Sulfate Alters Endothelial Junction-Associated Gene Expression

**Figure 5:**
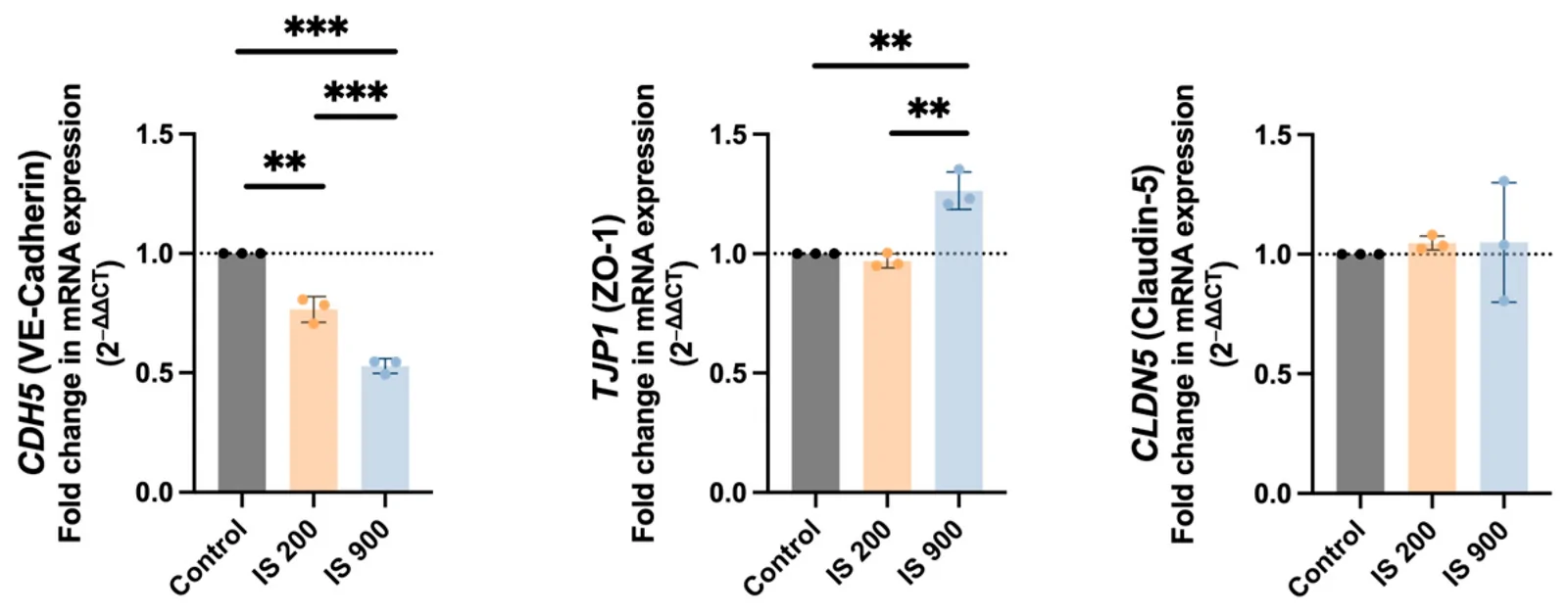
Relative mRNA expression levels of CDH5, TJP1, CLDN-5, in hCMEC/D3 cells following 48 h exposure to IS (200 and 900 µM) normalized to control using the 2^−ΔΔCt^ method. Data are presented as mean ± SD (n = 3 independent biological experiments). Statistical comparisons performed using a mixed-effect model with Šídák’s multiple-comparison test. The dashed horizontal line indicates the baseline control expression (fold change = 1). Statistical significance is indicated as ** *p* < 0.01, *** *p* < 0.001. Abbreviations: TJP1 (Tight junction protein 1), CDH5 (Cadherin-5), CLDN-5 (Claudin-5), IS (indoxyl sulfate).

IS exposure differentially affected endothelial junction transcripts. CDH5 expression was significantly downregulated following IS exposure, with greater reduction observed at IS 900 µM. TJP1 expression was significantly increased at IS 900 µM, but was unchanged at IS 200 µM. CLDN5 expression remained comparable to the control group at both IS concentrations (Figure 5).

### 2.7. Indoxyl Sulfate Alters the Secretome Profile (Exploratory Analysis)

**Figure 6:**
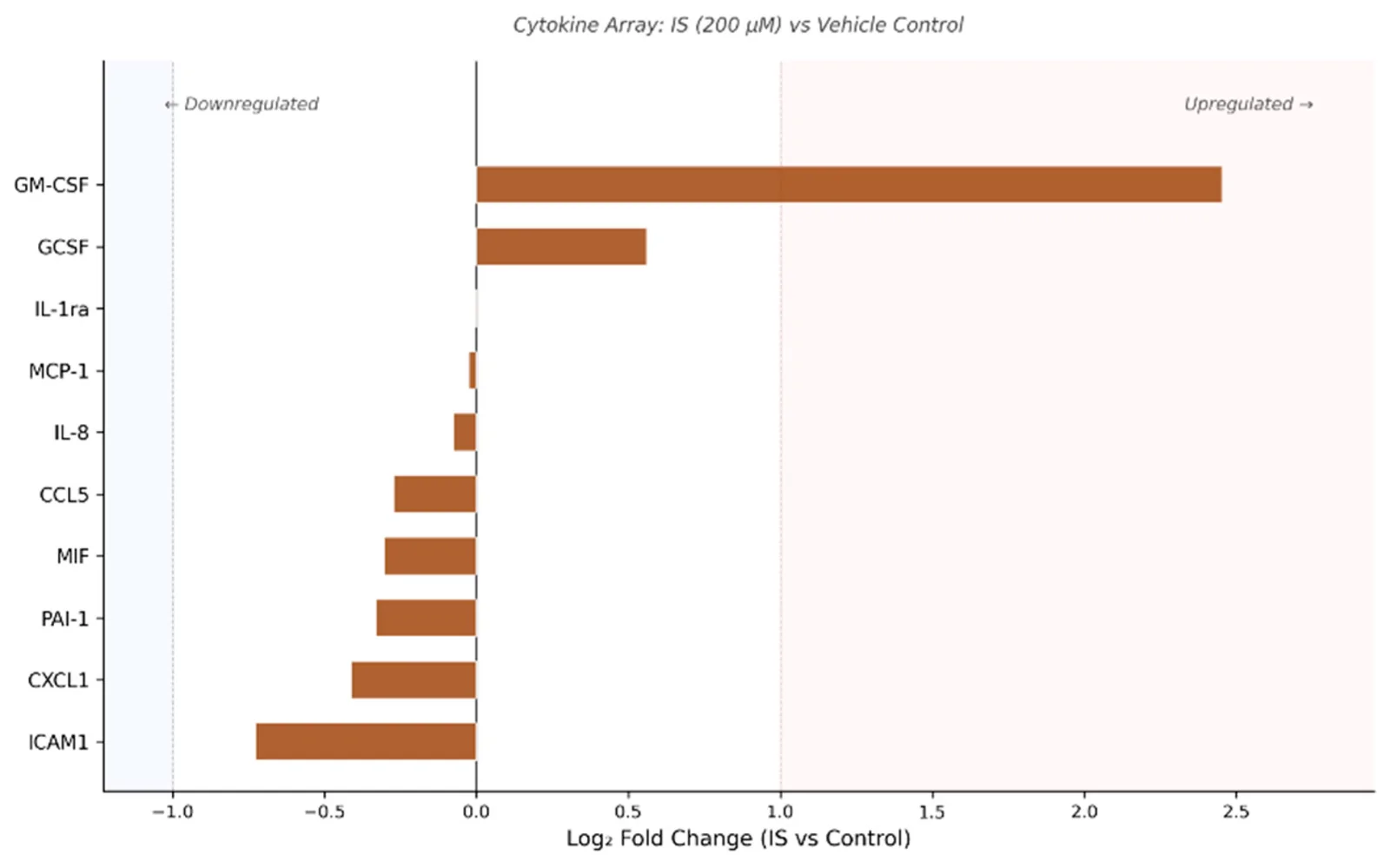
Secretome profile of the pooled supernatants of hCMEC/D3 cells exposed to IS 200 µM. Data are presented as Log_2_ fold-change relative to control, and the direction of the bars indicate the direction and magnitude of the cytokine alterations. Positive values indicate increased secretion, and negative values indicate reduced secretion compared to control. Pooled supernatant from n = 3 independent experiments; one analytical measurement for IS 200 µM and one for control. Abbreviations: GM-CSF (Granulocyte-Macrophage Colony-Stimulating-Factor), G-CSF (Granulocyte Colony-Stimulating-Factor), IL-1ra (Interleukin-1 Receptor Antagonist), MCP-1 (Monocyte Chemoattractant Protein-1), IL-8 (Interleukin-8), CCL-5 (C-C Motif Chemokine Ligand 5), PAI-1 (Plasminogen Activator Inhibitor-1), CXCL-1 (C-X-C Motif Chemokine Ligand 1), ICAM-1 (Intercellular Adhesion Molecule-1).

This cytokine profiling was performed on pooled supernatants from three independent experiments, yielding a single measurement per condition. This assay is therefore exploratory and descriptive, and is not supported by inferential statistics. Pooled supernatants of hCMEC/D3 cells treated with IS 200 µM revealed differential regulation of secreted factors relative to vehicle control. GM-CSF showed the most prominent increase, followed by G-CSF. Downregulated cytokines included ICAM1 (largest decrease), CCL-5, MIF, PAI-1, and CXCL1 (Figure 6).

### 2.8. Indoxyl Sulfate Increases SA-β-Galactosidase Activity

**Figure 7:**
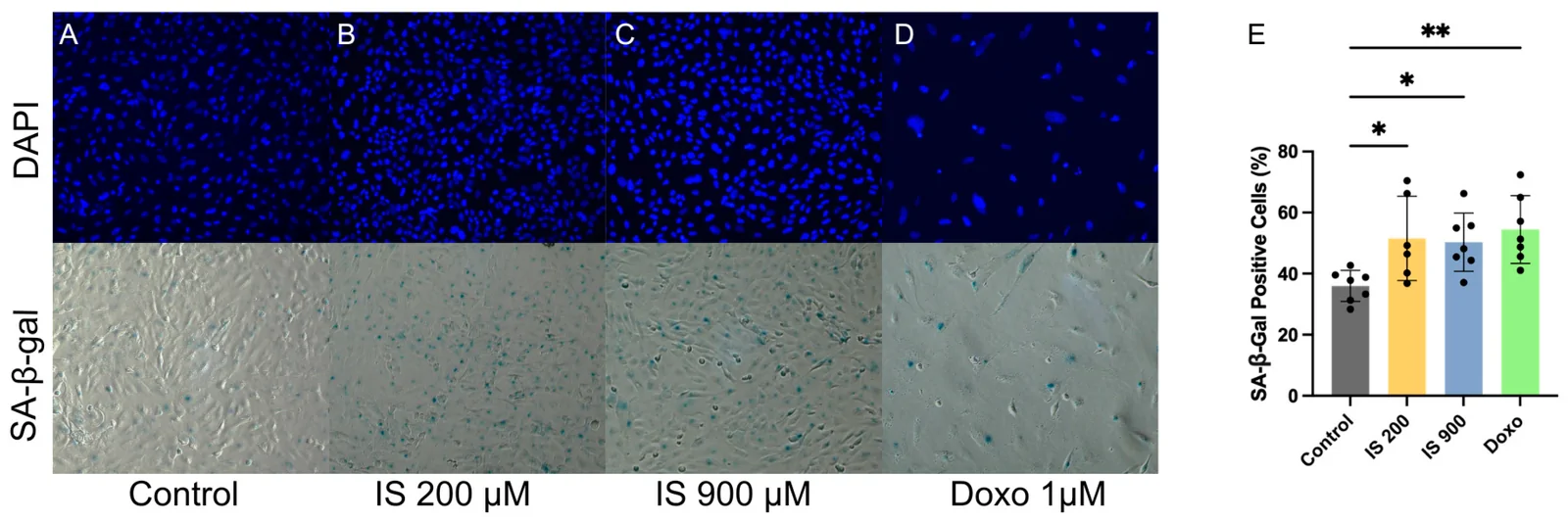
Representative images of hCMEC/D3 cells (**A**–**D**) treated for 48 h with IS 200 µM, IS 900 µM and Doxo 1µM (positive control). Upper panel shows DAPI nuclear staining, and lower panel shows SA-β-gal brightfield imaging, where blue-green cytoplasmic staining indicates SA-β-gal positive cells. Columns show untreated control, IS 200 µM, IS 900 µM, Doxorubicin. Scale bar: 100 µm (10× objective). (**E**) Quantification showed increased SA-β-gal positive cells compared to untreated control. Data presented as mean ± SD (n = 3 independent biological experiments). Statistical comparisons performed using a mixed-effect model with Šídák’s multiple-comparison test. Statistical significance at * *p* < 0.05, ** *p* < 0.01. Abbreviations: SA-β-gal (senescence associated beta-galactosidase), IS (indoxyl sulfate), Doxo (Doxorubicin).

To investigate whether IS promotes senescence-associated phenotypic changes, SA-β-gal activity was assessed following 48 h exposure to IS 200 µM and IS 900 µM. IS-treated cells demonstrated a significantly higher proportion of SA-β-gal-positive cells compared to untreated control (*p* < 0.01). A low level of baseline SA-β-gal positivity was observed in control cells, indicating inherent heterogeneity in the endothelial population. Doxorubicin (1 µM), an anthracycline-class genotoxic agent recognized for its ability to trigger senescent phenotype in cancerous and normal cell lines [21,22], was used as positive control for the induction of stress-induced premature senescence [21]. Doxorubicin readout showed increased SA-β-gal positivity and confirmed assay responsiveness (Figure 7).

## 3. Discussion

Our findings demonstrate that IS concentrations chosen on the basis of reported CKD-associated levels (200–900 µM) are associated with impaired BBB integrity in an in vitro human brain endothelial model without inducing overt cytotoxicity. IS exposure resulted in increased permeability, differential remodeling of endothelial junction components, robust AhR activation, selective upregulation of oxidative stress-associated genes, altered secretome profiles (exploratory), and increased SA-β-gal positivity consistent with a senescence-like endothelial phenotype.

Clinical relevance of the model: The IS concentrations used in this study were based on reported circulating IS concentrations in CKD and were intended to model clinically measured uremic IS exposure. Reported circulating IS levels vary substantially according to CKD stage, dialysis status, and whether total or free IS was measured [4,7,23,24,25,26,27,28,29]. This distinction is important because IS is a highly protein-bound uremic toxin, with more than 90% being bound to human serum albumin [30]. The 200 µM IS concentration used here approximates clinically reported total IS exposure in advanced CKD [23,24,31]. The 900 µM IS represents the upper-range values reported in literature [25], and is interpreted as a high-uremic-stress condition in our setup. In vitro, however, the relationship between IS exposure and the bioavailable IS fraction differs from plasma due to lower albumin concentrations, and CKD-related albumin modifications are not reproduced. Vanholder et al. [32] noted that insufficient albumin in experimental systems can result in disproportionately high free concentrations of protein-bound uremic toxins, which could potentially exaggerate biological responses. We therefore emphasize that our present in vitro model reflects cellular responses to nominal IS exposure under experimental conditions, rather than direct equivalence to circulating free IS concentrations in patients.

No cytotoxic effects were observed at these levels, consistent with findings in intestinal epithelial cells [26]. However, studies in human umbilical vein endothelial cells (HUVECs) have demonstrated reduced cell viability at similar concentrations [27]. This discrepancy likely reflects differences in metabolic and barrier properties between endothelial subtypes. Importantly, the preservation of cell viability in our model indicates that the observed functional changes reflect a dysregulated endothelial phenotype rather than cell loss, consistent with the chronic, sublethal nature of uremic toxin exposure in CKD.

Barrier disruption: IS exposure induced a sustained increase in endothelial permeability at both 24 and 48 h in our model. The similar magnitude of permeability observed at 200 and 900 µM suggests that barrier-disruptive mechanisms are activated already at moderate uremic concentrations, with limited additional impairment at higher levels within this timeframe. These findings are consistent with experimental evidence showing that declining renal function alters cerebral endothelial function [28], and that prolonged exposure to uremic toxins could induce behavioral changes in CKD animal models [18,33,34]. Accumulation of IS has also been associated with radio-imaging markers of BBB disruption [28]. The persistence of increased permeability over 48 h, despite baseline drift in controls conditions, indicates that IS exerts a sustained rather than transient effect on barrier integrity.

Junction remodeling: IS exposure significantly alters mRNA expression of junction-related genes, with downregulation of CDH5 and upregulation of TJP1 at higher IS concentration, while CLDN5 mRNA expression remained unchanged. The reduction in CDH5 is consistent with the potential destabilization of adherens junctions as CDH5 is a key regulator of endothelial cell–cell adhesion and its loss can compromise barrier integrity independently of tight junction alterations [35,36].

The increase in TJP1 at the higher 900 µM IS concentration may reflect junctional remodeling or a compensatory transcriptional response aimed at maintaining junctional scaffolding under stress, as reported in other endothelial and epithelial injury models [37]. In contrast, the absence of significant CLDN5 transcriptional changes, together with reduced claudin-5 immunostaining, suggests that IS-induced permeability changes may not be explained by transcriptional suppression of claudin-5 alone. Claudin-5 is regulated at multiple levels beyond transcription, including post-transcriptional regulation, internalization, and lysosomal or proteasomal degradation [38]. Moreover, claudin-5 barrier function depends not only on total abundance, but also on correct junctional localization and interaction with scaffolding proteins such as ZO-1, which links claudins to the actin cytoskeleton and supports tight junction organization [38,39]. The apparent discrepancy between unchanged CLDN5 mRNA and reduced claudin-5 protein signal may therefore reflect several protein-level mechanisms. Immunofluorescence intensity reflects not only protein abundance but also epitope masking, post-translational modification, or altered trafficking. These findings highlight the importance of a multilayered interpretation, as RNA abundance may not necessarily correlate with functional protein amount. Multiple post-transcriptional mechanisms regulate nascent mRNA along their trajectory from transcription to protein translation, including splicing and microRNA-mediated silencing via Dicer/RISC complex [40]. The present data are compatible with a model wherein barrier dysfunction involves subtle junctional reorganization, altered protein localization, or cytoskeletal mechanisms that may occur without robust changes in core tight junction transcripts’ abundance [41]. However, additional protein-level validation, including assessment of VE-cadherin, and/or ZO-1 abundance and localization, would help determine whether altered junctional protein organization contributes to this observed barrier dysfunction.

AhR signaling: Given that IS exposure did not cause overt cytotoxicity in our model, we examined whether IS concentrations within the range reported in CKD could induce transcriptional responses in hCMEC/D3 cells. A dose-dependent increase in CYP1A1, CYP1B1, and CYP1A2 expression was observed, indicating that IS activates canonical AhR-mediated xenobiotic metabolism gene expression in human brain endothelial cells. Similar induction of AhR target genes has been reported in peripheral endothelial models [12,18] and murine osteoclasts [42]. AhR-driven CYP induction is relevant to BBB pathology because cytochrome P450 enzymes can generate reactive metabolites that amplify oxidative stress in endothelial cells [10,29]. These observations are consistent with but do not establish a role for AhR activation in the downstream functional and gene transcript changes observed in this study. No AhR antagonist experiments were performed; therefore, the relationship between AhR pathway activation and BBB disruption in this model is interpreted as an association rather than a demonstrated causal mechanism.

Oxidative stress and inflammation: Reactive oxygen species (ROS) production and mitochondrial status were not directly assessed in this study; our discussion therefore refers to oxidative stress-associated transcriptional responses rather than demonstrated oxidative stress. IS exposure selectively altered these responses in hCMEC/D3 cells: NFE2L2 was significantly upregulated at both IS concentrations, and IDO1 was significantly increased at IS 900 µM, while SOD1 and NFKB1 expression remained unchanged. NFE2L2 upregulation suggests activation of an Nrf2-associated antioxidant stress-response program consistent with the known role of Nrf2 as a key regulator of cellular antioxidant defense under oxidative stress conditions [43,44]. The absence of corresponding SOD1 change indicates that IS did not drive a uniform transcriptional response across antioxidant defense genes. IDO1 upregulation supports selected inflammatory and AhR-associated downstream responses. Given the role of IDO1 in tryptophan–kynurenine immunomodulation downstream of AhR, this pathway warrants further investigation in studies with greater statistical power [45]. NFκB and Nrf2 are known to be reciprocally regulated, and an imbalance between the two could aggravate disease pathology in CKD [44]. The combination of NFE2L2 upregulation with unchanged NFKB1 in our model is more consistent with an early adaptive antioxidant response than with an established inflammatory phenotype at this timepoint. This pattern is in line with CKD being a state of chronic low-grade inflammation that may require longer exposure to fully manifest at the transcriptional level [46]. This early response interpretation is supported by reports of Nrf2 displaying a biphasic pattern during the course of CKD progression, rising in mild-to-moderate disease before declining to levels comparable with those of healthy individuals [47,48]. This is consistent with the possibility that antioxidant capacity may become repressed as CKD advances [43,49]. Additionally, in a comparable in vitro system, Colombo et al. reported that Nrf2 activation in IS-treated microvascular endothelial cells peaked before subsequently declining [20]. Taken together, our findings suggest that the Nrf2 upregulation observed at 48 h in our model may reflect an early, compensatory phase of the oxidative stress-associated response whose magnitude and direction likely depend on experimental timing and endpoint, and that could attenuate with more prolonged uremic toxin exposure.

Senescence: In this study, the term “senescence-like phenotype” is used to describe increased SA-β-gal positivity together with altered secretion of senescence-associated cytokines while acknowledging the absence of definitive evidence of stable cell-cycle arrest in our model. For senescence associated-genes, CDKN1A was significantly reduced at IS 900 µM, while CDKN2A remained unchanged. This pattern indicates that IS exposure was not accompanied by transcriptional activation of the canonical p21- or p16-associated cell-cycle arrest pathways, and therefore should not be interpreted as definitive evidence of established cellular senescence. This dissociation is not unprecedented: p21 regulation may follow a biphasic response in which moderate stress promotes transcriptional induction, while more severe damage may trigger proteasomal degradation [50,51]. In addition, p16 and p21 may mark distinct senescent subpopulations rather than sequential stages of a single program [52].

SA-β-gal positivity, while widely utilized as a senescence-associated marker, is not sufficient on its own as a definitive indicator of senescence. The assay detects β-galactosidase activity at pH 6, which has been attributed to lysosomal β-galactosidase; therefore, increased staining may also reflect increased lysosomal content or activity under cellular stress [53,54]. By consensus criteria, no single marker, including SA-β-gal, establishes senescence, and stable cell-cycle arrest was not assessed in this study. In endothelial cells, this senescence-like phenotype denotes enlarged, non-proliferating cells with increased SA-β-gal activity and a SASP-like secretory profile, consistent with stress-induced premature senescence [55]. Given that IS is associated with oxidative stress-related endothelial responses, a stress-induced premature senescence-like framework may be relevant for interpreting the observed phenotype in our study, but further validation is required.

Consistent with this interpretation, IS-treated cells exhibited increased SA-β-gal activity compared with untreated control, whereas doxorubicin positive control also showed a significant increase, confirming assay responsiveness. Exploratory secretome profiling revealed elevated levels of GM-CSF and G-CSF–cytokines previously associated with senescence-driven BBB dysfunction [56]. Taken together, our findings are therefore best interpreted as an IS-induced stress phenotype with features overlapping cellular senescence, including an altered secretory phenotype and SA-β-gal positivity, rather than fully established, cell-cycle-arrested senescence at this time point. This distinction may be particularly relevant to the framework of EVA paradigm in CKD, where early senescence-like endothelial changes may precede irreversible cell-cycle exit and accumulate during prolonged uremic exposure as the disease progresses [8,9,11].

Clinical implications: The observation that functional barrier disruption occurred in the absence of cytotoxicity and with relatively modest transcriptional changes in junction-associated genes suggests that early BBB injury in CKD may be subclinical and precede detectable cognitive decline. Cognitive impairment in CKD is frequently underrecognized and diagnosed at advanced stages of disease [12]. Existing experimental evidence links IS accumulation with BBB permeability and cognitive impairment and neuroinflammatory changes in animal models [18,33,57,58]. Clinical data in hemodialysis patients suggest higher serum levels in patients with impaired executive function [59]. However, whether circulating IS can independently predict longitudinal cognitive decline, or clinical translation of IS-lowering strategies remains uncertain. In the large randomized EPPIC trials, the oral charcoal adsorbent AST-120 did not significantly slow CKD progression when added to standard therapy in patients with moderate-to-severe CKD [60]. Moreover, the interpretation of circulating IS in dialysis populations is complicated by the distinction between total and free IS fractions, as only free IS is dialyzable, and post-dialysis rebound occurs between sessions, which may produce fluctuating rather than sustained clearance effects. Our findings support the hypothesis that uremic toxin-mediated EVA-related mechanisms such as oxidative stress-associated transcriptional responses, senescence-like phenotypic shifts, and junctional remodeling may occur in the cerebral microvasculature and may contribute to the neurovascular complications of CKD.

## 4. Strengths and Limitations

Our study provides a focused characterization of IS-associated barrier impairment in a human cerebral microvascular endothelial model. We integrated functional permeability with junctional, AhR-related, stress-associated, and senescence-associated readouts. The use of parallel viability assessment supports our interpretation of the permeability and transcriptional changes as sublethal endothelial impairment rather than the effect of overt cytotoxicity. Several limitations should likewise be acknowledged. The hCMEC/D3 monoculture model, although widely used as an in vitro BBB model, does not recapitulate the full structural and functional complexity of the neurovascular unit, which includes astrocytic endfeet, pericytes, and basement membrane. Transendothelial electrical resistance (TEER) measurements were not performed in the present study. Instead, barrier integrity was assessed using FITC-Dextran permeability, which measures paracellular influx. Our hCMEC/D3 culture conditions were closely aligned with those described by Tigro et al. [61], including the use of EndoGRO-MV complete medium and static transwell culture. Tigro et al. characterized TEER under these hCMEC/D3 conditions and reported low peak TEER values at approximately 32 Ω·cm^2^, while dextran permeability was lowest at the same point, supporting the formation of a functional low-permeability endothelial barrier despite low TEER values. Based on this closely comparable model characterization, FITC-Dextran was selected as the primary functional readout of paracellular permeability in the present study. Additionally, barrier maturation was also inferred based on previously published characterization of hCMEC/D3 monolayers [61,62,63,64]. However, we acknowledge the absence of parallel TEER measurements as a methodological limitation. Cells used in our study were from passages 30–33, which is within the range reported in previous hCMEC/D3 studies, with Weksler et al. reporting stable growth and endothelial marker characteristics until passage 35 [65]. Nevertheless, barrier properties in hCMEC/D3 cells can be influenced by passage number, culture duration and transwell conditions; hence, passage- or culture-related phenotypic drift cannot be fully excluded and should be considered when interpreting baseline permeability [64,65,66]. Gene expression analysis was performed at a single time point (48 h) and may not capture earlier or later transcriptional dynamics. In addition, SASP profiling was conducted at one concentration (200 µM) using pooled supernatants, which limits dose–response analysis and statistical inference for these endpoints. VE-cadherin and ZO-1 were assessed only at the transcript level in our model, and protein-level and localization studies for other junction proteins represent an important direction for future work. No pharmacological or nutritional inhibitors (e.g., AhR antagonists, Nrf2 inhibitors) were employed, precluding direct causal attribution of observed changes to specific signaling pathways. Finally, ROS production was not directly measured; thus, oxidative stress-associated changes are inferred from transcriptional responses.

## 5. Conclusions and Future Prospects

This study shows that exposure of human cerebral microvascular endothelial cells to the protein-bound uremic toxin IS is associated with impaired BBB integrity, altered endothelial junction-associated readouts, AhR-related transcriptional activation, oxidative stress-associated transcriptional responses, and senescence-like phenotypic features, all in the absence of overt cytotoxicity. Because AhR inhibition or knockdown was not performed, these relationships should be interpreted as associative rather than causal. These findings support a potential mechanistic framework linking uremic toxin exposure and cerebrovascular endothelial dysfunction, and position IS-associated BBB injury within the broader framework of EVA in CKD. The causal mechanistic relationships among the pathways involved remain to be investigated.

Future research should address several open questions emerging from these findings. First, pharmacological or nutritional inhibition of AhR or Nrf2 pathways would help determine whether the transcriptional responses are causally linked to barrier disruption. Second, direct assessment of ROS production and redox dynamics would clarify the role of oxidative stress. Third, time-course experiments encompassing both earlier and later timepoints are required to delineate the temporal dynamics of Nrf2--NFκB signaling and the emergence of a senescence-like phenotype. Fourth, future studies incorporating multicellular human BBB models with astrocytes and pericytes, including iPSC-derived neurovascular-unit and organ-on-chip systems, are needed to test whether these endothelial-monoculture findings persist within a complete neurovascular unit. Finally, because the uremic milieu contains multiple protein-bound solutes that may act synergistically, the individual and combined effects of other toxins, including TMAO and phenylacetylglutamine (PAG), on BBB integrity and cerebrovascular dysfunction warrant further investigation.

## 6. Materials and Methods

### 6.1. Cell Culture and Treatments

Human cerebral microvascular endothelial cells (hCMEC/D3) (CLU512, Cedarlane, ON, Canada) were cultured in collagen-coated (collagen type I, rat tail, 08-115, Sigma Aldrich, Merck KGaA, Darmstadt, Germany) flasks containing EndoGRO complete medium. The complete medium was composed of EndoGro basal media (SCME-BM, Sigma Aldrich) and EndoGRO MV supplements (SCME004-S, Sigma Aldrich) with 2% EndoGRO LS Supplement, 5 ng/mL rHEGF, 10 mM L-glutamine, 1 µg/L hydrocortisone, 0.75 U/mL heparin sulfate, 50 µg/mL ascorbic acid, 5% FBS, 1% penicillin–streptomycin (15140-122, Gibco, Thermo Fisher Scientific, Waltham MA, USA), and 1 ng/mL human basic fibroblast growth factor (F0291-25UG, Sigma Aldrich). Cells were incubated at 37 °C in a humidified atmosphere with 5% CO_2_. Culture medium was changed every 2–3 days, and cells from passages 30–33 were used for experiments. To minimize passage-related confounding, all treatment conditions within each biological replicate were performed using cells of the same passage number and compared against concurrent controls. The hCMEC/D3 cells were grown and maintained in standard growth medium for 5 days to allow for monolayer formation and junctional maturation before IS exposure. Previous reports indicated that optimal barrier characteristics in hCMEC/D3 are observed between days 4 and 6 after seeding [63,65,66]. All experiments were performed on three separate occasions.

The treatment groups included: control (untreated), and indoxyl sulfate (I3875-1G, Sigma Aldrich) 200 and 900 µM. A two-concentration design was used for this initial characterization of IS-associated endothelial responses in hCMEC/D3 cells, comparing lower uremic exposure with a severe uremic burden [4,24,25,31]. Because IS is highly protein-bound in vivo, these in vitro concentrations should be interpreted as total IS exposures under experimental culture conditions rather than direct equivalents of circulating free IS concentrations in patients. This protocol aimed to establish our BBB injury model using the uremic toxin IS associated with CKD.

### 6.2. Cell Viability

hCMEC/D3 cells were seeded in duplicate in collagen type I rat-tail-coated 48-well plates (92048, Thermo Fisher Scientific) at a density of 50,000 cells/cm^2^ and exposed to different concentrations of IS (200 and 900 µM) for 48 h. Prior to treatment, the medium was replaced with serum-free medium overnight and on treatment day replaced with low-serum media (1% FBS) containing IS. Technical duplicates were pooled prior to counting. Viable cells were quantified by Trypan blue (11538886, Gibco, Thermo Fisher Scientific) exclusion using an automated cell counter (Countess, Invitrogen, Thermofisher). Viability is reported as the mean percentage of viable cells per pooled condition in each independent experiment.

### 6.3. Permeability Assay

Transwell inserts (PET membrane, 0.4 µm pore, 83.3932.041 Sarstedt, AG & Co., KG, Nümbrecht, Germany) were coated with collagen type I rat-tail (5%) and seeded with hCMEC/D3 cells, which were allowed to attach overnight and grown for 5 days. Cells were exposed for 48 h to IS (200 or 900 µM). Cytochalasin D (2.5 µM), an actin-disrupting agent known to alter tight junction organization and increase permeability [67], served as positive control for maximal barrier disruption. Vehicle-treated control inserts received medium only.

FITC-Dextran 4 kDa (Cat#4013, Chondrex Inc., Woodinville, WA, USA) was added to the apical chamber together with the toxin treatments. At the start of the assay (T0), the apical compartment received 400 µL medium containing a final concentration of 4 µg/mL (1 µM) FITC-Dextran 4 kDa. The basolateral compartment received 800 µL medium without FITC-Dextran. Plates were incubated at 37 °C in a humidified 5% CO_2_ incubator. At 24 h, 150 µL of basolateral media was collected and replaced with an equal volume of fresh medium; the apical chamber was left undisturbed to maintain the FITC-concentration gradient. At 48 h, all the apical and basolateral media were collected and recovered volumes measured. Samples were stored at −20 °C protected from light until analysis.

For FITC-Dextran quantification, thawed samples were centrifuged briefly and fluorescence measured on a solid black 96-well plate (137101, Thermo Fisher Scientific, Roskilde, Denmark) and a reading was taken by microplate reader (Ex/Em 490/560) (Infinite M1000, Tecan, Männedorf, Switzerland). Concentrations were determined by linear interpolation from a fresh FITC-Dextran standard curve prepared in the same incubation medium. Apparent permeability was calculated as follows:

P_app_ (cm/s) = dQ/dT/A∗C_0_

where: dQ/dT (µg/s) is the rate of mass influx of 4 kDa FITC-Dextran; A (cm^2^) is the surface area of the insert; and C_0_ (µg/mL) is the initial concentration of the 4 kDa FITC-Dextran in the apical compartment. For each endpoint, dQ/dT was calculated using the cumulative amount of FITC-Dextran recovered in the basolateral chamber divided by the elapsed time from T0 to the sampling point. Thus, dQ/dT was calculated using 86,400 s for the 24 h endpoint and 172,800 s for the 48 h endpoint. Vehicle-only controls were run concurrently at each timepoint, and IS-induced permeability was interpreted relative to these concurrent controls. No additional baseline subtraction was applied because all treatment groups and controls were measured within the same assay run and were subject to the same assay-level variation or drift. The values for permeability were additionally expressed relative to the positive control Cytochalasin D-treated cells for visualization of the effect size in relation to the maximal barrier disruption in our setup.

### 6.4. Immunofluorescence

IS-treated hCMEC/D3 cells grown in transwell inserts were fixed in 4% paraformaldehyde for 10 min and washed three times with PBS. Permeabilization was done with 0.3% Triton for 20 min, followed by blocking with 10% goat serum at room temperature. Claudin-5 primary antibody (1:200, CSB-PA005507LA01HU, Cusabio Technology, Nordic Biosite AB, Stockholm, Sweden) was incubated overnight at 4 °C in PBS buffer containing 0.3% Triton X-100 and 5% goat serum. After three PBS washes, secondary antibody (1:300, A11012, Invitrogen, Thermo Fisher Scientific) diluted in 5% goat serum was applied for 1 h at room temperature in the dark. Inserts were washed thrice with PBS, counterstained with DAPI for 10 min, and then washed three times with PBS before mounting with an anti-fade medium. Fluorescence intensity was measured from four random fields per insert per condition in each independent experiment. For statistical analysis, field measurements were averaged per insert, and testing was performed on biological replicate means.

### 6.5. RNA Extraction, cDNA Synthesis and qPCR

Total RNA was isolated after 48 h of treatment using the RNeasy kit (Qiagen, Hilden, Germany) per manufacturer’s guidelines. RNA concentration and purity were assessed using a Nanodrop spectrophotometer (Nanodrop 2000c, Thermo Fisher Scientific, Wilmington, DE, USA).

Reverse transcription was conducted using the Maxima first strand cDNA Synthesis Kit with dsDNase (Cat#K1671, Thermo Fisher Scientific). The reaction was prepared in nuclease-free PCR tubes by combining 10X dsDNase buffer, dsDNase, template RNA (1 μg), nuclease-free water, 5X reaction mix, and Maxima enzyme mix to achieve a final reaction volume of 20 μL. For quality control, reverse transcriptase (RT)-negative controls were prepared by excluding the reverse transcriptase (Maxima enzyme mix), while no-template controls (NTCs) were generated by including all reagents except the RNA template. The thermal cycling conditions were as follows: 10 min at 25 °C, 30 min at 50 °C, and 5 min at 85 °C. The synthesized cDNA was stored at −80 °C until further analysis.

Quantitative PCR (qPCR) was performed using PowerTrack SYBR Green Master Mix (Thermo Fisher Scientific) (Bio-Rad CFX-384, Hercules, CA, USA). Each reaction mixture (20 μL) contained 2 μL cDNA (10 ng), 10 μL PowerTrack SYBR Green master mix, 1.6 μL of each primer (400 nM final concentration), nuclease-free water, and 0.5 μL of sample buffer. Melting curve analysis was conducted to confirm the specificity of each amplification reaction. Primer sequences are listed in Supplementary Table S1.

Genes of interest included those involved in AhR signaling, oxidative stress/inflammation, senescence and endothelial junctions; CYP1A1, CYP1B1 and CYP1A2 (AhR target genes); NFE2L2 (encoding Nrf2), SOD1, NFKB1, and IDO1; CDKN1A (encoding p21) and CDKN2A (encoding p16) (senescence regulators); and CLDN5 (claudin-5), TJP1 (ZO-1), and CDH5 (VE-cadherin) (junction-associated genes). Gene expression was quantified using threshold cycle (Ct) values from each sample. Expression levels of target genes were normalized to the mean of two reference housekeeping genes: glyceraldehyde 3-phosphate dehydrogenase (GAPDH) and beta-actin (ACTB).

### 6.6. Secretome Profiling

Supernatants from three independent experiments of hCMEC/D3 exposed to IS 200 µM were pooled to assess SASP using a human cytokine array (ARY005B, R&D Systems, Minneapolis, MN, USA), which was performed according to the manufacturer’s instructions. The SASP profiling was performed at IS 200 µM only to assess whether a lower exposure level could already promote secretion of inflammatory mediators. This approach was designed to explore whether secretory changes may precede overt senescence-associated staining. As our array incubation represents a single analytical measurement per condition, the results were reported descriptively as fold-change relative to control.

### 6.7. SA-β-Galactosidase Assay

hCMEC/D3 cells were seeded in 24-well plates (83.3922, Sarstedt) (20,000 cells/well), allowed to attach overnight, and incubated with IS 200 and IS 900 µM for 48 h. The assay was performed using a commercially available kit (Cat#9860, Cell Signaling Technology, Beverly, MA, USA) according to the manufacturer’s instructions. The senescent cells were visualized under a light microscope, where blue-positive cells were counted and expressed as a proportion of total cells per field. Doxorubicin (1 µM), a known inducer of DNA damage and endothelial senescence [21], served as a stress-associated positive control to confirm assay responsiveness.

### 6.8. Statistical Analysis

Independent biological replicates were used as the unit of analysis. Technical replicates within the same biological replicate were averaged before statistical testing. The number of biological replicates for each experiment is indicated in the corresponding figure.

For qPCR experiments, relative gene expression was calculated using the 2^−ΔΔCt^ method. Statistical analysis for qPCR was performed on ΔCt values, while fold-change values are shown for visualization.

For experiments with matched treatment conditions across biological replicates, statistical comparisons were performed using a mixed-effect model for repeated-measure data, with treatment condition as the fixed effect and the experimental replicate as the matched/repeated factor. Pairwise comparisons between treatment groups were adjusted using Šídák’s multiple-comparison test.

Secretome profiling was performed on pooled supernatants from independent experiments generating only one analytical measurement per condition. Therefore, data were reported descriptively without inferential statistical testing.

Data are presented as mean ± SD unless otherwise stated. A *p*-value < 0.05 was considered statistically significant. All statistical analyses were performed using GraphPad Prism (v 9.0., Boston, MA, USA).

## Data Availability

The original contributions presented in this study are included in this article. Further inquiries can be directed to the corresponding authors.

## Supplementary Materials

The following supporting information can be downloaded at: https://www.mdpi.com/article/10.3390/toxins18080334/s1, Figure S1: Permeability result of hCMEC/D3 monolayers. Comparison of absolute permeability (Papp) of control, IS 200 μM, IS 900 μM, and cytochalasin D positive control; Table S1: Primer sequences of genes used for qPCR.

## Abbreviations

The following abbreviations are used in this manuscript:

CKD	Chronic kidney disease
BBB	Blood–brain barrier
IS	Indoxyl sulfate
EVA	Early vascular aging
SASP	Senescence-associated secretory phenotype
hCMEC/D3	Human cerebral microvascular endothelial cells
AhR	Aryl hydrocarbon receptor
SA-β-gal	Senescence-associated beta galactosidase
CYP	Cytochrome
NFE2L2/Nrf2	Nuclear factor, erythroid 2 like 2
IDO1	Indoleamine 2,3-dioxygenase 1
NFKB1/NFκB	Nuclear factor kappa B subunit 1
SOD1	Superoxide dismutase 1
CDKN1A/p21	Cyclin-Dependent Kinase Inhibitor 1A
CDKN2A/p16	Cyclin-Dependent Kinase Inhibitor 2A
GM-CSF	Granulocyte-Macrophage Colony-Stimulating Factor
G-CSF	Granulocyte Colony-Stimulating Factor
CDH5	Cadherin 5
TJP1/ZO-1	Tight junction Protein 1/Zonula Occludens-1
CLDN5	Claudin 5
FITC	Fluorescein isothiocyanate
ROS	Reactive oxygen species
