# Supplementary material for: Indoxyl Sulfate-Mediated Blood–Brain Barrier Damage in Chronic Kidney Disease
Source: Toxins (Basel). 2026 Aug 1;18(8):334. doi: 10.3390/toxins18080334 (PMC13517478; doi:10.3390/toxins18080334)
Supplement: Supplementary file 1 [file toxins-18-00334-s001.zip › toxins-4404376-supplementary.pdf]

Supplementary Materials: Indoxyl Sulfate-Mediated Blood–Brain Barrier Damage in Chronic Kidney Disease

Leah Hernandez, Camillo Tancredi Strizzi, Miriam Rosina, Angelina Schwarz, Nina Kronqvist, Samsul Arefin, Peter Stenvinkel, Karolina Kublickiene on behalf of MOMENTUM Consortium

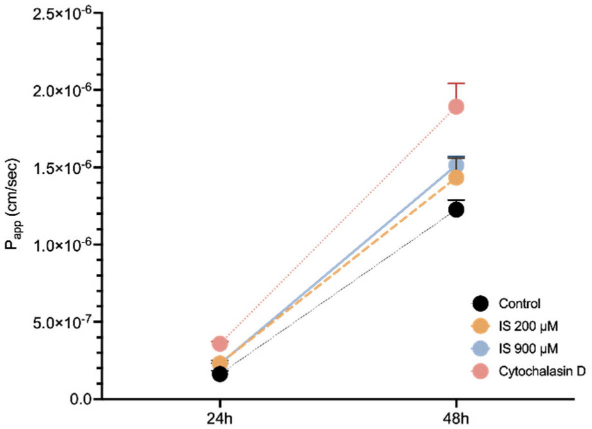

Supplement Figure S1. Permeability result of hCMEC/D3 monolayers. Comparison of absolute permeability (P_{app}) of control, IS 200 µM, IS 900 µM, and cytochalasin D positive control.

Supplement Table S1. Primer sequences of genes used for qPCR.

Gene	Forward Primer (5'-3')	Reverse Primer (5'-3')
GAPDH	GAGTCAACGGATTTGGTCGT	GACAAGCTTCCCGTTCTCAG
ACTB	GGACCTGACTGACTACCTCAT	CGTAGCACAGCTTCTCCTTAAT
Cyp1a1	ACATGCTGACCCTGGGAAAG	GGTGTGGAGCCAATTCGGAT
Cyp1b1	AAGTTCTTGAGGCACTGCGAA	GGCCGGTACGTTCTCCAAAT
Cyp1a2	CTTCGCTACCTGCCTAACCC	GACTGTGTCAAATCCTGCTCC
NRF2	TCTGACTCCGGCATTTCACT	GGCACTGTCTAGCTCTTCCA
SOD1	CGAGTTATGGCGACGAAGGC	GGACCTGCACTGGTACAGCC
NFkB	ATCCCATCTTTGACAATCGTGC	CTGGTCCCGTGAAATACACCTC
IDO1	GTGGGCTTTGCTCTGCCAAA	GCGCTGTGACTTGTGGTCTG
p21	AGGTGGACCTGGAGACTCTCAG	TCCTCTTGGAGA AGATCAGCCG
p16	CTCGTGCTGATGCTACTGAGGA	GGTCGGCGCAGTTGGGCTCC
CLDN-5	GAGGCGTGCTCTACCTGTTT	TAAAACTCGCGGACGACAATG
ZO-1	CGAGCGATCTCATAAACTTCGT	CCTCGGAAACCCATACCAG
VE-Cadherin	ACAAGGACATAACACCACGAA	CCGTGTTATCGTGATTATCCGTGA
